# RESILIENCE AND PERCEIVED ORAL HEALTH AMONG OLDER CHINESE AMERICAN IMMIGRANTS: THE ROLE OF SOCIAL SUPPORT

**DOI:** 10.1093/geroni/igae098.1429

**Published:** 2024-12-31

**Authors:** Nan Jiang, Wei Zhang, Bei Wu

**Affiliations:** Tsinghua University, Beijing, Beijing, China (People’s Republic); University of Hawai‘i at Mānoa, Honolulu, Hawaii, United States; New York University, New York, New York, United States

## Abstract

Improving the oral health of older immigrants is a critical public health priority in the United States. However, the role of children’s support in this context has received limited attention. This study aims to investigate the relationship between support from adult children and perceived oral health among both foreign-born and U.S.-born Chinese Americans. Additionally, we explore the mediating role of resilience. We analyzed data from a sample of 377 Chinese American older adults aged 55 or older in Honolulu, Hawai’i. Utilizing structural equation models, we compared self-rated oral health and oral health problems between foreign-born and U.S.-born Chinese Americans. For foreign-born participants, greater emotional support from children was directly associated with better perceived oral health. Furthermore, this emotional support was indirectly linked to perceived oral health through resilience. In contrast, for U.S.–born Chinese Americans, financial support from children was directly related to worse perceived oral health. This study provides evidence for resilience pathways that connect social support from children to older immigrants’ oral health. Encouraging adult children in Chinese immigrant families to educate their older relatives about oral diseases and dental care can enhance the oral health-related quality of life for older Chinese immigrants.

